# Active Compensation Technology for the Target Measurement Error of Two-Axis Electro-Optical Measurement Equipment

**DOI:** 10.3390/s24041133

**Published:** 2024-02-09

**Authors:** Lintao Lan, Fangwu Hua, Fang Fang, Wei Jiang

**Affiliations:** 1 State Key Laboratory of Intelligent Manufacturing Equipment and Technology, School of Mechanical Science and Engineering, Huazhong University of Science and Technology, Wuhan 430074, China; lanlintao_717@126.com; 2 Huazhong Institute of Electro-Optics, Wuhan National Laboratory for Optoelectronics, Wuhan 430223, Chinafangrifang@163.com (F.F.)

**Keywords:** target measurement error, error compensation technology, least square, AKF, RBFNN

## Abstract

For two-axis electro-optical measurement equipment, there are many error sources in parts manufacturing, assembly, sensors, calibration, and so on, which cause some random errors in the final measurement results of the target. In order to eliminate the random measurement error as much as possible and improve the measurement accuracy, an active compensation technique for target measurement error is proposed in this paper. Firstly, the error formation mechanism and error transfer model establishment of the two-axis electro-optical measurement equipment were studied, and based on that, three error compensation and correction methods were proposed: the least square (LS)-based error compensation method, adaptive Kalman filter(AKF)-based error correction method, and radial basis function neural network (RBFNN)-based error compensation method. According to the theoretical analysis and numerical simulation comparison, the proposed RBFNN-based error compensation method was identified as the optimal error compensation method, which can approximate the random error space surface more precisely, so that a more accurate error compensation value can be obtained, and in order to improve the measurement accuracy with higher precision. Finally, the experimental results proved that the proposed active compensation technology was valid in engineering applicability and could efficiently enhance the measurement accuracy of the two-axis electro-optical measurement equipment.

## 1. Introduction

The two-axis electro-optical measurement equipment is extensively utilized in astronomical navigation, fire control tracking, landing guidance, and other domains. Its target measurement accuracy serves as a critical technical indicator and directly impacts navigation precision, target hit rate, aircraft landing accuracy, and more. Therefore, enhancing the equipment’s target measurement accuracy holds significant importance in achieving a higher mission success rate. Particularly in time-sensitive tasks, there is an increasing demand for improved equipment measurement accuracy. Consequently, greater attention has been devoted to research and technological advancements aiming at enhancing the measurement accuracy of the two-axis electro-optical measurement equipment.

The two-axis electro-optical measurement equipment is a sophisticated opto-mechatronics device, encompassing various error sources in parts manufacturing, assembly, sensor measurement, and equipment calibration. These error sources ultimately impact the accuracy of the measurements obtained from the equipment. Therefore, it is imperative to analyze how these error sources transfer and subsequently affect the target’s measurement accuracy in order to explore error compensation techniques. Several scholars have conducted research on system error analysis. Liu et al. developed a tracking error model of a solar dish concentrator system based on the rigid body motion theory, and analyzed the effect of the azimuth tilt error on the tracking performance [1]. Li established the position error model and motion error model of the turn-milling combined NC machine tool, and identified the important sensitive error terms by evaluating the influence weights of each error term [2]. Wang constructed the TCP error model of the 3-DOF parallel spindle head using the geometric errors, and got six critical geometric errors [3]. Fang built the geometric error model of the 6-axis welding equipment with 36 geometric error components based on Lie theory, and analyzed the sensitivity of geometric errors by numerical simulation, but no experimental proof was obtained [4]. There are also some studies on error modeling and error sensitivity analysis for five-axis machine tool. A volumetric error model with multiple geometric errors was established based on the multi-body system method, and the corresponding sensitivity analysis method to identify the vital geometric error was proposed [5,6]. In addition, a new PIGEs (position-independent geometric errors) identification model based on DMM (differential motion matrices) was constructed, and the minimum set of PIGEs can be easily found [7]. Furthermore, Yin proposed a programmable identification method to decouple the PDGEs (position dependent geometric errors) and PIGEs, which greatly optimized the error identification method [8]. Different from the above error sensitivity analysis methods, which were carried out in the global coordinate system, the quantitative interval sensitivity analysis method was presented, and the key geometric errors were identified at different intervals [9]. Furthermore, for the robot, Feng used a homogeneous transformation matrix to develop the mapping relationship between the end-effector position error and geometric source errors within the serial mechanism kinematic chains, and studied the kinematics of the spatial serial mechanism with a large number of geometric errors [10]. Li used a full matrix complete differential method to construct the error model of a cable-driven parallel robot, the results confirmed that the cable length errors and pulleys’ geometric errors should be given higher priority in design [11]. San built the error mapping model for the parallel mechanism part of the hybrid robot by using the closed-loop vector method and the first-order perturbation method, and identified the most significant factors affecting the robot’s end posture error, but no error compensation [12]. All of the above studies are based on the kinematics of serial mechanism with multiple geometric errors, and the error model is established through a hierarchical transfer relationship. The primary objective is to analyze the sensitivity of each error source and identify significant error terms with greater influence weight, in order to propose design and operational recommendations. However, there has been limited discussion on the strategy for error compensation.

For the two-axis electro-optical measurement equipment, the design or operation suggestions for some important error terms cannot meet the requirements of improving the target measurement accuracy. Therefore, it is necessary to find a method that actively compensate the measurement error in real time. Fortunately, there has been a growing body of literature on error compensation that primarily focuses on enhancing system performance. Firstly, some error compensation methods have been studied for improving the machining accuracy of parts; these methods inspired people to explore improving the accuracy for other systems [13,14,15,16]. Zhou analyzed the error model of LED chip visual localization systems by the Monte Carlo method, and found that the position acquisition error was the largest error source of the system positioning accuracy error. Then, the corresponding error compensation model was obtained by the LS method, but the number of error sources analyzed was relatively small [17]. In the optical system, increased attention has been paid to the pointing error. An error compensation model was established by analyzing the error sources of the pointing error for the shipboard photoelectric telescope, aiming at the multicollinearity of the model parameters. Moreover, a stepwise regression method was proposed to compensate the repetitive systematic errors, but the nonlinear errors were not covered [18]. A linear pointing error model of the optical communication terminal was established by reformulating the linear equations with dependent variables represented by star measurement data, and the parameter vector was determined by the LS method; however, the parameter vector has no clear physical meaning and the enhancement of pointing accuracy is limited [19]. Similarly, the distortion model of a ground-based telescope is given with no apparent reason, and the fitting coefficients are determined by a simulated annealing algorithm; moreover, the correction effect is basically the same as the physical model [20]. The pointing error of a mirror normal can greatly affect the optical axis pointing accuracy; Zhao has built a digital calibration for the mirror normal pointing error by using the quaternion mathematical method [21]. Different from the general method, the error model of 3D laser scanning is established based on BP neural networks, which links the mathematical error influencing factor and measurement deviation; the final measurement is optimized by the correction of point cloud and the global calibration optimization based on the error model [22] Researchers have proposed some error compensation algorithms for specific error sources, and achieved good results. However, limited attention has been paid to random error. In fact, system accuracy would be enhanced more efficiently if the random error is also taken into account in the error compensation method.

In this paper, both the specific and random error sources of the equipment are taken into consideration. By establishing an error model and conducting error analysis, an active error compensation technology is proposed to enhance measurement accuracy. Firstly, an error transfer model was established based on the equipment’s working principle, and the simulated target measurement error was analyzed. Subsequently, three error compensation or correction methods were proposed. According to the simulation comparison result, the RBFNN-based error compensation method was identified as the optimal approach. Finally, experimental results validated the effectiveness of the proposed active error compensation technique.

## 2. The Measurement Error Model Establishment and Analysis

**Figure 1:**
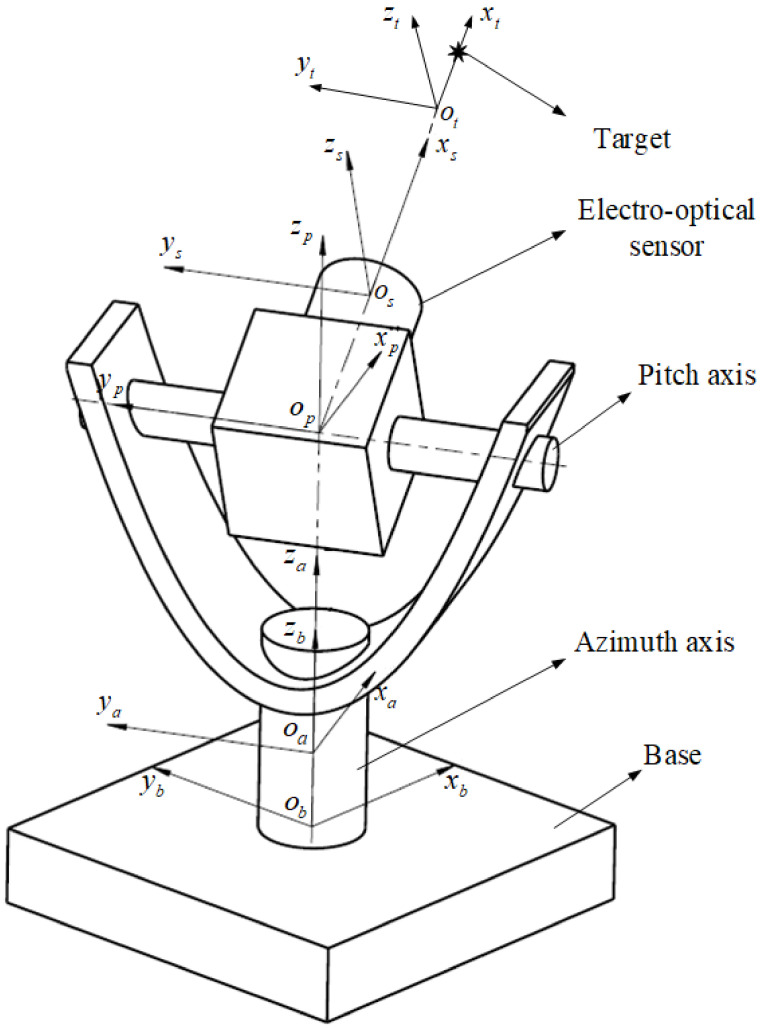
The structure schematic diagram of two-axis electro-optical measurement equipment.

Figure 1 shows the target measurement principle diagram of the two-axis electro-optical measurement equipment. The target coordinate system $o_{t}-x_{t}y_{t}z_{t}$ is fixed to the target, the target vector can be expressed as ${\left[\begin{array}{ccc} 1 & 0 & 0 \end{array}\right]}^{T}$, the sensor coordinate system $o_{s}-x_{s}y_{s}z_{s}$ is fixed to the sensor, the pitch coordinate system $o_{p}-x_{p}y_{p}z_{p}$ is fixed to the pitch axis, the azimuth coordinate system $o_{a}-x_{a}y_{a}z_{a}$ is fixed to the azimuth, and the base coordinate system $o_{b}-x_{b}y_{b}z_{b}$ is fixed to the base. When measuring the target, the azimuth coordinate system rotates an azimuth angle α relative to the base coordinate system, and the sensor coordinate system rotates a pitch angle β relative to the pitch axis coordinate system, so that the sensor coordinate system coincides with the target coordinate system, and the spatial position coordinates of the target are obtained under the base coordinate system. However, due to the parts machining and assembly errors, rolling bearing errors, angle measurement errors, servo control errors, target extraction errors, and other error sources, there are some errors in the measurement results, described in the following steps from the target measurement process to explore the impact of each link error on the measurement results.

**Figure 2:**
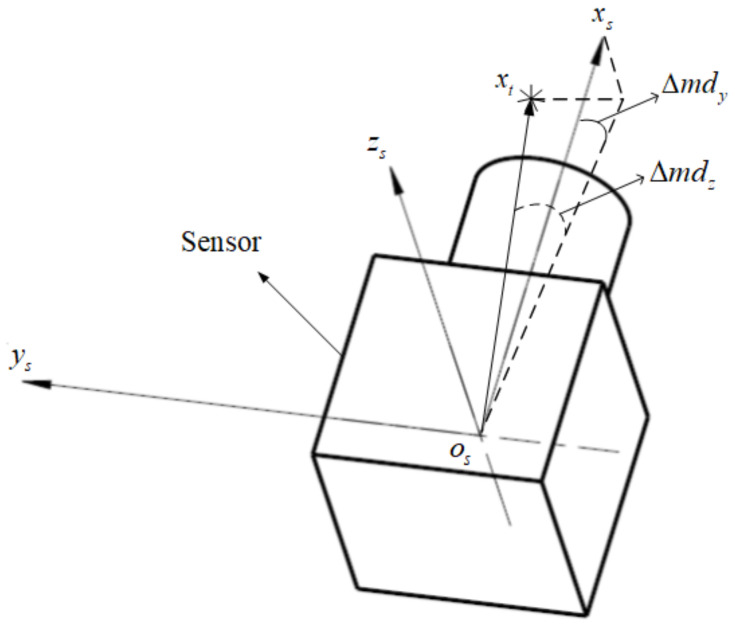
The target extraction errors.

Due to the target image extraction errors, the LOS does not really point to the target centroid, but has a certain error angle with the target centroid. As shown in Figure 2, the coordinate axis $x_{t}$ points to the target centroid, the coordinate axis $x_{s}$ represents the LOS, the errors $\Delta md_{y}$ and $\Delta md_{z}$ between them are random errors, and the transformation matrix from $o_{t}-x_{t}y_{t}z_{t}$ to $o_{s}-x_{s}y_{s}z_{s}$ is represented as $T_{t}^{s}$.

(1) $$T_{t}^{s}=\left[\begin{array}{ccc} \cos(\Delta md_{y}) & 0 & \sin(\Delta md_{y})\\ 0 & 1 & 0\\ -\sin(\Delta md_{y}) & 0 & \cos(\Delta md_{y}) \end{array}\right]\cdot \left[\begin{array}{ccc} \cos(\Delta md_{z}) & -\sin(\Delta md_{z}) & 0\\ \sin(\Delta md_{z}) & \cos(\Delta md_{z}) & 0\\ 0 & 0 & 1 \end{array}\right]$$

**Figure 3:**
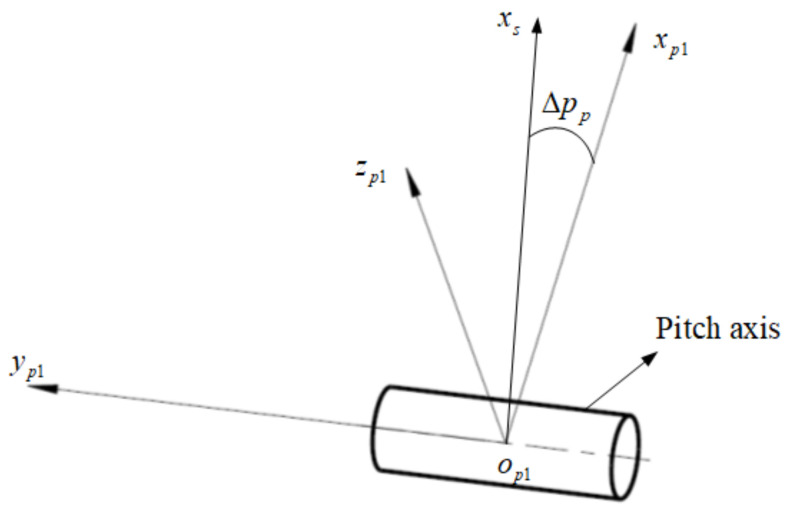
The perpendicularity error of the sensor installation.

Due to the sensor assembly errors, there is a certain perpendicularity error between the LOS and the pitch axis. In Figure 3, the coordinate axis $x_{p1}$ is the ideal axis perpendicular to the pitch axis, the perpendicularity error $\Delta p_{p}$ is uniformly distributed, and the transformation matrix from $o_{s}-x_{s}y_{s}z_{s}$ to $o_{p1}-x_{p1}y_{p1}z_{p1}$ is represented as $T_{s}^{p1}$.

(2) $$T_{s}^{p1}=\left[\begin{array}{ccc} \cos\Delta p_{p} & -\sin\Delta p_{p} & 0\\ \sin\Delta p_{p} & \cos\Delta p_{p} & 0\\ 0 & 0 & 1 \end{array}\right]$$

**Figure 4:**
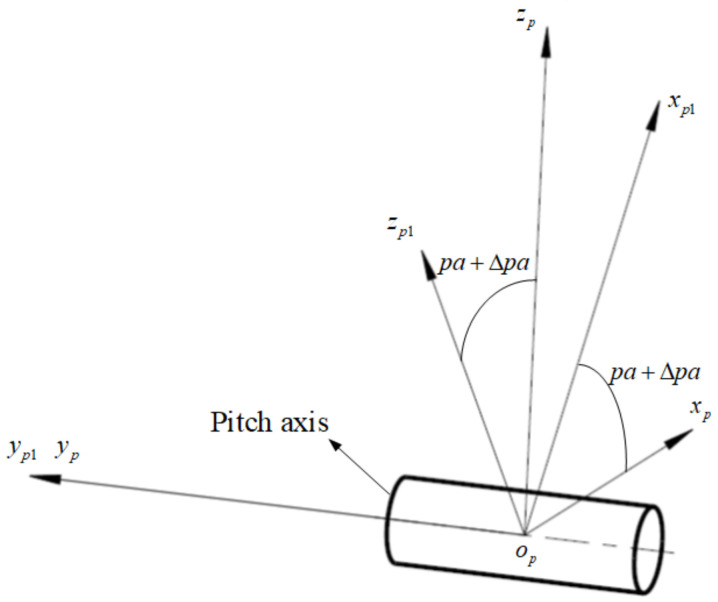
The pitch angle measurement error.

The pitch angle pa+Δpa of the LOS relative to the pitch coordinate system is measured by the angle measuring element mounted in the pitch axis, where pa is the true pitch angle, Δpa is the angle measuring error caused by electrical and data truncation reasons, and the error is a random distribution error. According to Figure 4, the transformation matrix from $o_{p1}-x_{p1}y_{p1}z_{p1}$ to $o_{p}-x_{p}y_{p}z_{p}$ is represented as $T_{p1}^{p}$:

(3) $$T_{p1}^{p}=\left[\begin{array}{ccc} \cos(pa+\Delta pa) & 0 & -\sin(pa+\Delta pa)\\ 0 & 1 & 0\\ \sin(pa+\Delta pa) & 0 & \cos(pa+\Delta pa) \end{array}\right]$$

**Figure 5:**
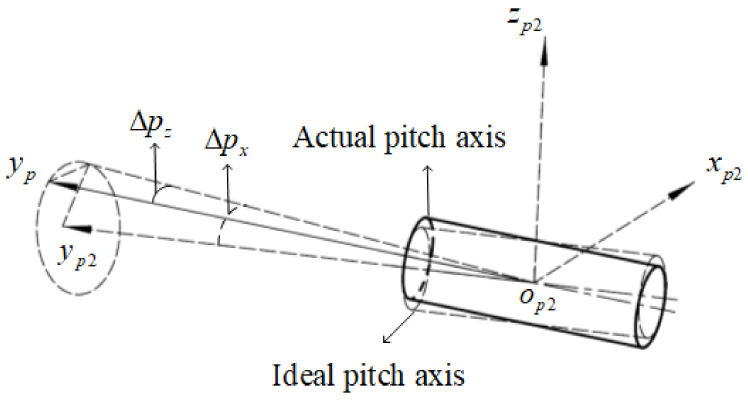
The pitch axis wobble errors.

Due to the rolling bearing clearance, assembly pre-tighten, and so on, there is a certain wobble when the pitch axis rotates. As shown in Figure 5, the coordinate axes $y_{p}$ and $y_{p2}$ are the actual pitch axis center and the ideal pitch axis, respectively, the wobble errors $\Delta p_{x}$ and $\Delta p_{z}$ are the random distribution errors, and the transformation matrix from $o_{p}-x_{p}y_{p}z_{p}$ to $o_{p2}-x_{p2}y_{p2}z_{p2}$ is represented as $T_{p}^{p2}$ in Equation (4).

(4) $$T_{p}^{p2}=\left[\begin{array}{ccc} 1 & 0 & 0\\ 0 & \cos\Delta p_{x} & -\sin\Delta p_{x}\\ 0 & \sin\Delta p_{x} & \cos\Delta p_{x} \end{array}\right]\left[\begin{array}{ccc} \cos\Delta p_{z} & -\sin\Delta p_{z} & 0\\ \sin\Delta p_{z} & \cos\Delta p_{z} & 0\\ 0 & 0 & 1 \end{array}\right]$$

**Figure 6:**
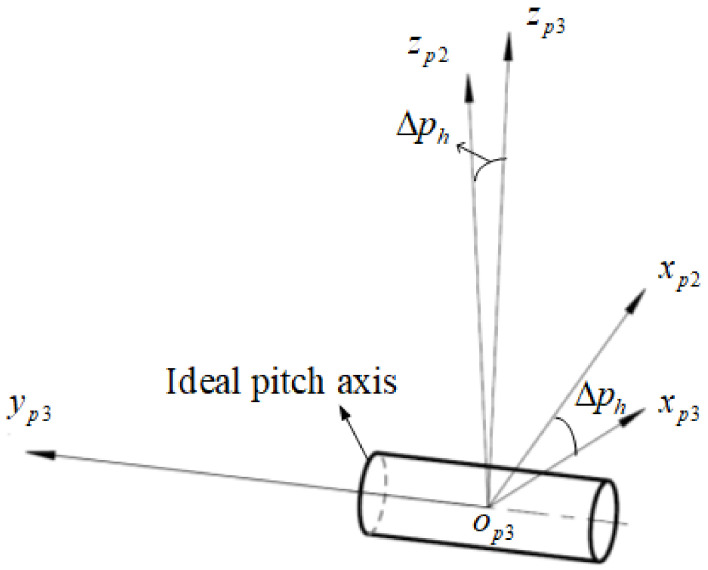
The pitch horizontal zero error.

The pitch angle measurement is based on the horizontal zero position. As shown in Figure 6, there is a certain electrical zero error $\Delta p_{h}$ in the horizontal zero position obtained by calibration, and this error is a uniform distributed. The transformation matrix is represented as $T_{p2}^{p3}$:

(5) $$T_{p2}^{p3}=\left[\begin{array}{ccc} \cos\Delta p_{h} & 0 & -\sin\Delta p_{h}\\ 0 & 1 & 0\\ \sin\Delta p_{h} & 0 & \cos\Delta p_{h} \end{array}\right]$$

**Figure 7:**
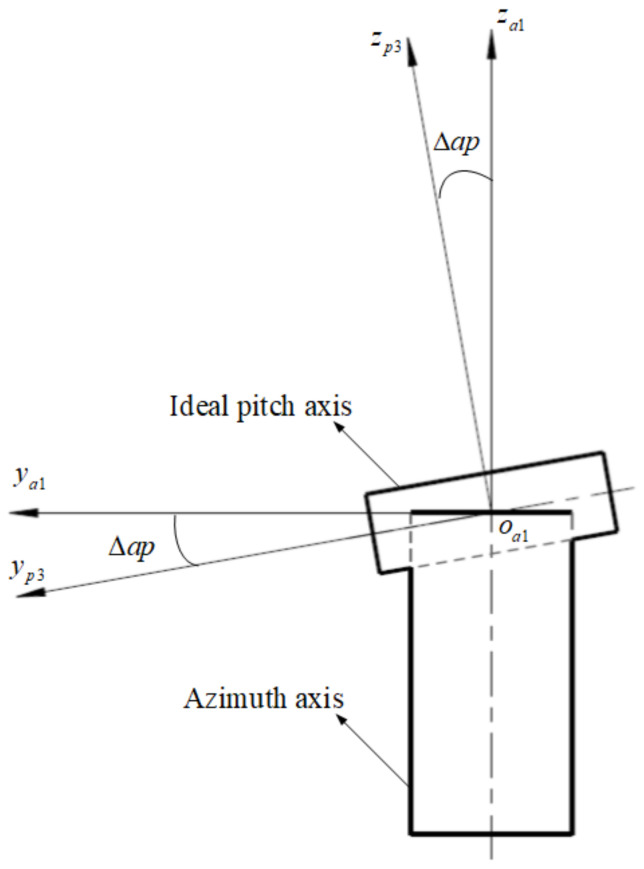
The perpendicular error between the pitch and the azimuth axes.

The pitch axis and the azimuth axis are two independent rotation axes, and when assembled together, there is inevitably a uniform distributed perpendicular error Δap between them; as shown in Figure 7, the transformation matrix from $o_{p3}-x_{p3}y_{p3}z_{p3}$ to $o_{a1}-x_{a1}y_{a1}z_{a1}$ is represented as $T_{p3}^{a1}$:

(6) $$T_{p3}^{a1}=\left[\begin{array}{ccc} 1 & 0 & 0\\ 0 & \cos\Delta ap & \sin\Delta ap\\ 0 & -\sin\Delta ap & \cos\Delta ap \end{array}\right]$$

**Figure 8:**
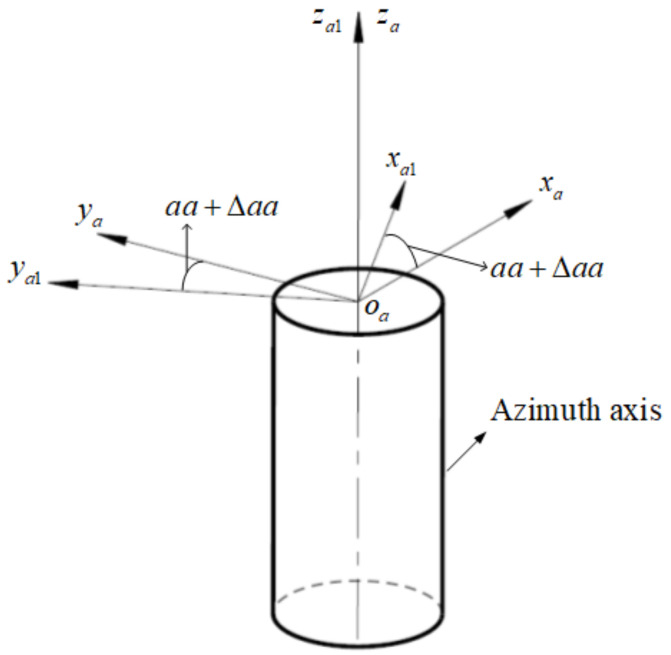
The azimuth angle measurement error.

Same as the pitch axis, the azimuth rotation angle measured is aa+Δaa, where aa is the true value, Δaa is the angle measuring error caused by electrical and data truncation reasons, and the error is random distributed. According to Figure 8, the transformation matrix from $o_{a1}-x_{a1}y_{a1}z_{a1}$ to $o_{a}-x_{a}y_{a}z_{a}$ is represented as $T_{a1}^{a}$:

(7) $$T_{a1}^{a}=\left[\begin{array}{ccc} \cos(aa+\Delta aa) & -\sin(aa+\Delta aa) & 0\\ \sin(aa+\Delta aa) & \cos(aa+\Delta aa) & 0\\ 0 & 0 & 1 \end{array}\right]$$

**Figure 9:**
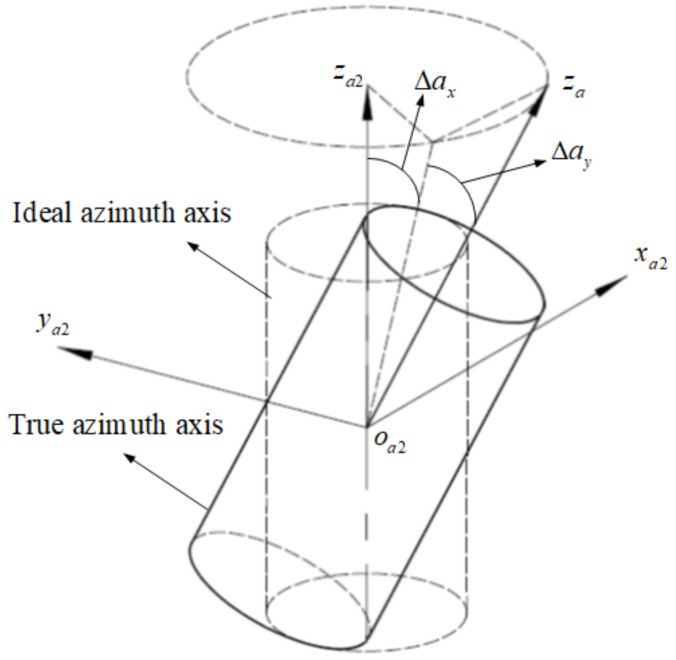
The azimuth axis wobble errors.

Same as the pitch axis, due to the rolling bearing clearance, assembly pre-tighten, and so on, there is a certain wobble when the azimuth axis rotates. As shown in Figure 9, the coordinate axes $z_{a}$ and $z_{a2}$ are the actual azimuth axis center and the ideal azimuth axis, respectively, the wobble $\Delta a_{x}$ and $\Delta a_{y}$ are random distributed, and the transformation matrix from $o_{a}-x_{a}y_{a}z_{a}$ to $o_{a2}-x_{a2}y_{a2}z_{a2}$ is represented as $T_{a}^{a2}$:

(8) $$T_{a}^{a2}=\left[\begin{array}{ccc} 1 & 0 & 0\\ 0 & \cos\Delta a_{x} & -\sin\Delta a_{x}\\ 0 & \sin\Delta a_{x} & \cos\Delta a_{x} \end{array}\right]\left[\begin{array}{ccc} \cos\Delta a_{y} & 0 & \sin\Delta a_{y}\\ 0 & 1 & 0\\ -\sin\Delta a_{y} & 0 & \cos\Delta a_{y} \end{array}\right]$$

**Figure 10:**
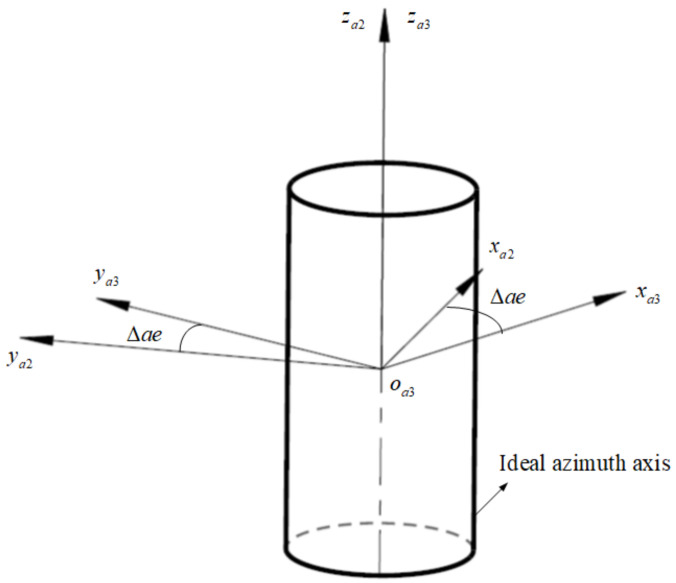
The azimuth zero position error.

Same as the pitch axis, the azimuth angle measurement is based on azimuth zero position. As shown in Figure 10, there is a certain electrical zero error in the azimuth zero position obtained by calibration, and this error is uniform distributed. The transformation matrix from $o_{a2}-x_{a2}y_{a2}z_{a2}$ to $o_{a3}-x_{a3}y_{a3}z_{a3}$ is represented as $T_{a2}^{a3}$:

(9) $$T_{a2}^{a3}=\left[\begin{array}{ccc} \cos\Delta ae & -\sin\Delta ae & 0\\ \sin\Delta ae & \cos\Delta ae & 0\\ 0 & 0 & 1 \end{array}\right]$$

**Figure 11:**
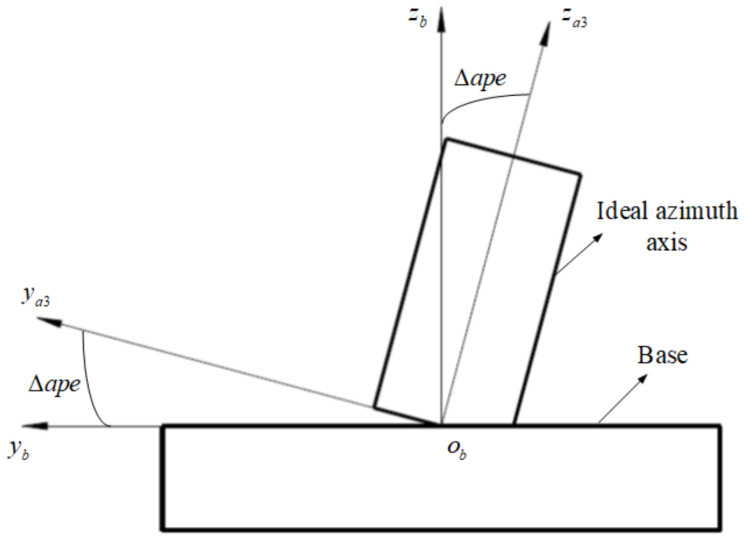
The equipment installation perpendicular error.

During the installation of the equipment, due to the flatness and parallelism of the base and other reasons, there is a certain perpendicular error Δape between the azimuth axis and the base, and the error is uniform distributed. As shown in Figure 11, the transformation matrix from $o_{a3}-x_{a3}y_{a3}z_{a3}$ to $o_{b}-x_{b}y_{b}z_{b}$ is represented as $T_{a3}^{b}$:

(10) $$T_{a3}^{b}=\left[\begin{array}{ccc} 1 & 0 & 0\\ 0 & \cos\Delta ape & -\sin\Delta ape\\ 0 & \sin\Delta ape & \cos\Delta ape \end{array}\right]$$

As mentioned above, in the actual target measurement, due to the existence of multiple error sources, the measured value of the target vector after a series of coordinate transformations from the target coordinate system to the base coordinate system is represented as:

(11) $$\left[\begin{array}{c} x\\ y\\ z \end{array}\right]=T_{a3}^{b}T_{a2}^{a3}T_{a}^{a2}T_{a1}^{a}T_{p3}^{a1}T_{p2}^{p3}T_{p}^{p2}T_{p1}^{p}T_{s}^{p1}T_{t}^{s}\left[\begin{array}{c} 1\\ 0\\ 0 \end{array}\right]$$

Converting the measured values of the unit target vector above from the Cartesian coordinate system to the spherical coordinate system, the azimuth and pitch angles are represented as:

(12) $$\left\{\begin{array}{c} \alpha =\mathrm{atan}(\frac{y}{x})\\ \beta =\mathrm{asin}(z)\;\; \end{array}\right.$$

If there is no serial error sources above, the theoretical measurement value of the target should be ${\left[\begin{array}{cc} aa & pa \end{array}\right]}^{T}$; then, the measurement error of the target is represented as:

(13) $$\left\{\begin{array}{c} \Delta \alpha =\alpha -aa\\ \Delta \beta =\beta -pa \end{array}\right.$$

According to the errors measured during the actual design and assembly of a two-axis electro-optical measurement equipment, the statistical results are shown in Table 1.

In addition to the error sources considered above, there are still some uncertain factors causing measurement errors in actual target measurement, such as wind load, atmospheric turbulence, etc. the target measurement errors caused by these uncertain factors can be set as follows:

(14) $$\Delta \alpha_{r}={\left(0.001\alpha \right)}^{2}+0.03\sin\left(2.0\alpha \right)$$

(15) $$\Delta \beta_{r}={\left(0.001\beta \right)}^{2}+0.03\sin\left(3.0\beta \right)$$

**Figure 12:**
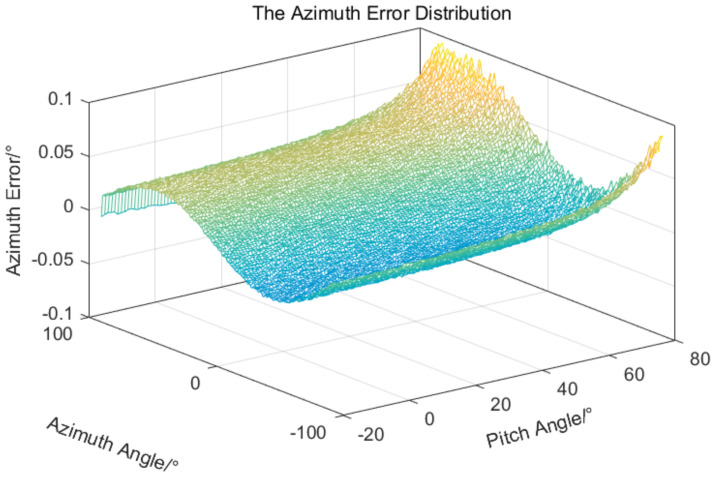
The azimuth measurement error distribution.

**Figure 13:**
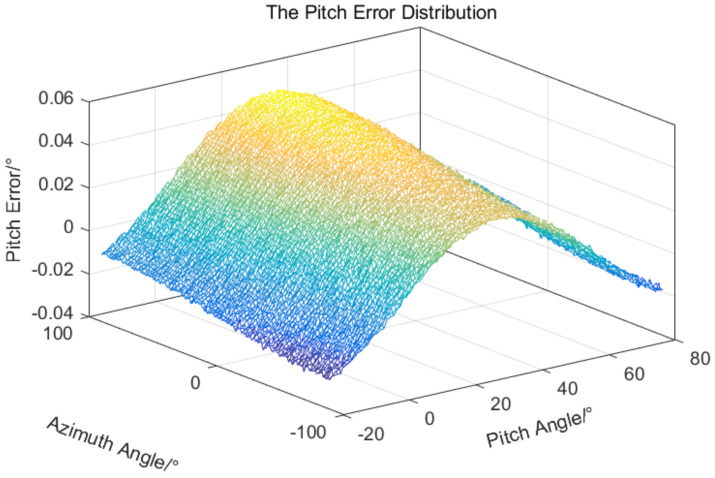
The pitch measurement error distribution.

The error data in Table 1 and the errors in Equations (14) and (15) are used to simulate the target measurement errors of a two-axis electro-optical measurement equipment; the results are shown in Figure 12 and Figure 13.

As can be seen from the above simulation results, due to the existence of systematic errors and random errors, the overall target measurement errors distribution is a spatial free-form surface distribution, and the error surface presents a random distribution of convex and uneven. Eliminating or reducing this error distribution is a technical challenge.

## 3. The Measurement Error Compensation and Correction Methods

From the theoretical derivation and simulation results presented in the previous section, it is evident that the measurement error of the equipment arises from a combination of multiple error sources and exhibits significant nonlinear variations with changes in azimuth and pitch angles. By employing the concept of least square method, an optimal error source space vector can be identified based on several sets of measurement data. Subsequently, the corresponding error value can be calculated using the measured azimuth and pitch values to compensate for measurement errors, thereby establishing an LS-based error compensation method. Similarly, by eliminating oscillating error values around the true measurement value, a more accurate measurement result can be obtained. The AKF algorithm proves effective in filtering out glitches present in measurement data; hence, an AKF-based error correction method may yield superior outcomes. Moreover, if we are able to identify this definite strong nonlinear mapping relationship and accurately determine its associated error value according to measured values, higher levels of measurement accuracy can be achieved. In theory, the RBFNN can approximate any nonlinear function with infinite accuracy, and therefore, utilizing an RBFNN-based error compensation method may lead to improved results.

### 3.1. LS-Based Error Compensation Method

This method takes all the above errors as unknown quantities, the LS method is used to solve the optimal error parameters, and compensates directly in the target measurement results finally. For this purpose, Equation (11) needs to be turned into a display expression. Since the error components values are relatively small, they can be considered in mathematical operation as follows:

(16) Δ·Δ≈0,sin(Δ)≈Δ,cos(Δ)≈1

Substitute Equation (16) into Equations (11) and (13), and then:

(17) $$\left\{\begin{array}{c} \Delta \alpha =x_{1}+x_{2}/\cos\beta +x_{3}\tan\beta -x_{4}\tan\beta \cos\alpha -x_{5}\tan\beta \sin\alpha \\ \Delta \beta =x_{4}\sin\alpha -x_{5}\cos\alpha +x_{6}\;\;\;\;\;\;\;\;\;\;\;\;\;\;\;\;\;\;\;\;\;\;\;\;\;\;\;\;\;\;\;\;\;\;\;\;\;\;\;\;\;\;\;\;\;\;\;\;\;\; \end{array}\right.$$

where $x_{1}=\Delta p_{z}+\Delta ae+\Delta aa$, $x_{2}=\Delta p_{p}+\Delta md_{z}$, $x_{3}=\Delta ap-\Delta p_{x}$, $x_{4}=\Delta a_{x}+\Delta ape$, $x_{5}=\Delta a_{y}$, and $x_{6}=\Delta p_{h}+\Delta pa-\Delta md_{y}$ are the unknown parameters to be solved.

By measuring multiple targets with known position, the measured values and corresponding measurement errors can be obtained, and the overdetermined equation can be constructed to solve the unknown parameters:

(18) $$\left[\begin{array}{cccccc} 1 & 1/\cos\beta_{1} & \tan\beta_{1} & -\tan\beta_{1}\cos\alpha_{1} & -\tan\beta_{1}\sin\alpha_{1} & 0\\ 0 & 0 & 0 & \sin\alpha_{1} & -\cos\alpha_{1} & 1\\ 1 & 1/\cos\beta_{2} & \tan\beta_{2} & -\tan\beta_{2}\cos\alpha_{2} & -\tan\beta_{2}\sin\alpha_{2} & 0\\ 0 & 0 & 0 & \sin\alpha_{2} & -\cos\alpha_{2} & 1\\ \vdots & \vdots & \vdots & \vdots & \vdots & \vdots \\ 1 & 1/\cos\beta_{n} & \tan\beta_{n} & -\tan\beta_{n}\cos\alpha_{n} & -\tan\beta_{n}\sin\alpha_{n} & 0\\ 0 & 0 & 0 & \sin\alpha_{n} & -\cos\alpha_{n} & 1 \end{array}\right]\left[\begin{array}{c} x_{1}\\ x_{2}\\ x_{3}\\ x_{4}\\ x_{5}\\ x_{6} \end{array}\right]=\left[\begin{array}{c} \Delta \alpha_{1}\\ \Delta \beta_{1}\\ \Delta \alpha_{2}\\ \Delta \beta_{2}\\ \vdots \\ \Delta \alpha_{n}\\ \Delta \beta_{n} \end{array}\right]$$

The equation above can be expressed as A·X=B, and using the LS method, the optimal error parameters can be obtained according to the following formulation:

(19) $$X={\left(A^{T}A\right)}^{-1}A^{T}B$$

By substituting the optimal error parameters into Equation (17), the measurement errors of a certain target measurement can be obtained. Then, the measurement results can be compensated by subtracting the measurement errors from the measurement values, expressed as:

(20) $$\left[\begin{array}{c} \alpha_{\mathrm{LS}}\\ \beta_{LS} \end{array}\right]=\left[\begin{array}{c} \alpha \\ \beta \end{array}\right]-{\left[\begin{array}{cc} 1 & 0\\ 1/\cos\beta & 0\\ \tan\beta & 0\\ -\tan\beta \cos\alpha & \sin\alpha \\ -\tan\beta \sin\alpha & -\cos\alpha \\ 0 & 1 \end{array}\right]}^{T}\cdot X$$

### 3.2. AKF-Based Error Correction Method

The AKF can be adopted to estimate the azimuth and pitch angles of the target measurement, which can filter out some systematic errors and random errors, and the more accurate target measurement values can be obtained.

According to Newton’s kinematics theory, the discrete state space equation of the system can be expressed as:

(21) $$\left\{\begin{array}{c} x_{k}=Tx_{k-1}+Iu_{k-1}+W_{k-1}\\ z_{k}=Mx_{k}+V_{k}\;\;\;\;\;\;\;\;\;\;\;\;\;\; \end{array}\right.$$

where $T=\left[\begin{array}{cccc} 1 & \Delta t & 0 & 0\\ 0 & 1 & 0 & 0\\ 0 & 0 & 1 & \Delta t\\ 0 & 0 & 0 & 1 \end{array}\right]$, $I=\left[\begin{array}{cc} 0.5\Delta t^{2} & 0\\ \Delta t & 0\\ 0 & 0.5\Delta t^{2}\\ 0 & \Delta t \end{array}\right]$; $x_{k}={\left[\begin{array}{cccc} \alpha_{k} & \omega_{k} & \beta_{k} & \gamma_{k} \end{array}\right]}^{T}$ is the state vector of the system at *k* moment, which consists of azimuth angle, azimuth velocity, pitch angle, and pitch velocity; T is the state transition matrix; I is the control matrix; Δt is the sampling interval; $u_{k-1}={\left[\begin{array}{cc} a_{k-1} & b_{k-1} \end{array}\right]}^{T}$ is the input matrix at moment k−1; $W_{k-1}$ is the process noise, and $W_{k-1}∼N\left(0,Q_{k-1}\right)$; $z_{k}={\left[\begin{array}{cc} \theta_{k} & \varphi_{k} \end{array}\right]}^{T}$ represents the measured value, which consists of measured azimuth and pitch angles; M is the measurement matrix; and $V_{k}$ is the measuring noise, and $V_{k}∼N\left(0,R_{k}\right)$.

The AKF method is used to estimate the measured azimuth and pitch angles; the specific algorithm steps are as follows:

Step 1: Prediction

Predicted state estimate: ${\hat{x}}_{k}^{-}=T{\hat{x}}_{k-1}+Iu_{k-1}$;

Priori covariance matrix: $P_{k}^{-}=TP_{k-1}T^{T}+Q_{k-1}$.

Step 2: Correction

Kalman gain: $K_{k}=P_{k}^{-}H^{T}{\left({\mathrm{HP}}_{k}^{-}H^{T}+R_{k}\right)}^{-1}$;

Measurement innovation: $d_{k}=z_{k}-{M\hat{x}}_{k}^{-}$;

Posteriori state estimate: ${\hat{x}}_{k}={\hat{x}}_{k}^{-}+K_{k}d_{k}$;

Residual: $\varepsilon_{k}=z_{k}-M{\hat{x}}_{k}$;

Posteriori covariance matrix: $P_{k}=P_{k}^{-}-K_{k}{\mathrm{HP}}_{k}^{-}$;

Measurement covariance update: ${\hat{R}}_{k}=\frac{1}{m}\sum_{i=0}^{m-1}\varepsilon_{k-i}{\varepsilon_{k-i}}^{T}+H_{k}P_{k}{H_{k}}^{T}$;

State covariance update: ${\hat{Q}}_{k-1}=K_{k}\frac{1}{m}\sum_{i=0}^{m-1}d_{k-i}{d_{k-i}}^{T}{K_{k}}^{T}$

After the above calculation, the optimal estimate of the system is ${\left[\begin{array}{cc} \alpha_{ACF} & \beta_{ACF} \end{array}\right]}^{T}$.

### 3.3. RBFNN-Based Error Compensation Method

The target measurement error distribution of the two-axis electro-optical measurement equipment is a random free-form surface. If a function can be found to fit or approximate the random free-form error surface, the measurement accuracy will be greatly improved. In theory, the RBFNN can approach any nonlinear function with infinite accuracy, so it is a better choice to use the RBFNN to approximate the error distribution. For this, the RBFNN is established as below.

**Figure 14:**
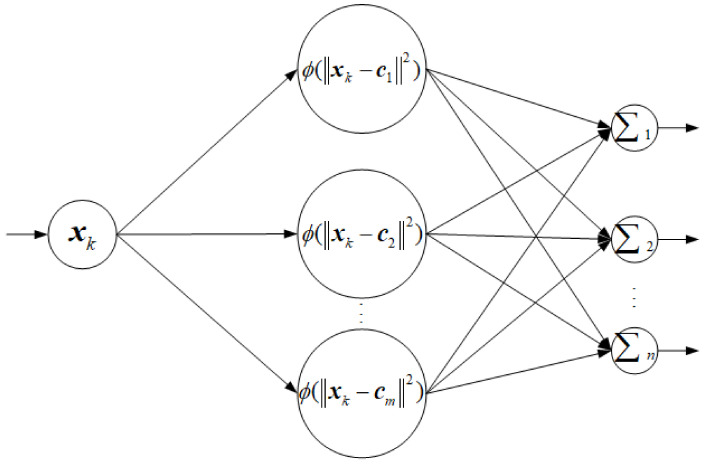
The RBFNN for approximating the error distribution.

As shown in Figure 14, the RBFNN consists of three layers of forward neural network, namely input layer, hidden layer, and output layer, respectively. The input layer $x_{k}$ is the measured value, i.e., the measured azimuth and pitch angles ${\left[\begin{array}{cc} \alpha & \beta \end{array}\right]}^{T}$; the hidden layer consists of *m* nonlinear neuron functions, which maps the linear input layer to the nonlinear space; and the last layer is the error output layer, which is a linear combination of the output of all the hidden layer neurons. According to the RBFNN established for error estimation, the final estimate of measurement error can be expressed as:

(22) $${\hat{y}}_{i}=W_{i}^{T}h_{k}=\sum_{j=1}^{m}w_{i,j}h_{k,j}$$

where ${\hat{y}}_{i}$ is the output errors estimated, ${\hat{y}}_{1}=\Delta \alpha$ and ${\hat{y}}_{2}=\Delta \beta$ are the target measurement azimuth and pitch errors estimated, respectively; $W_{i}$ is the corresponding weight coefficient, $W_{i}={\left[\begin{array}{cccc} w_{i,1} & w_{i,2} & \cdots & w_{i,m} \end{array}\right]}^{T}$; and $h_{k}$ is the neuron function of the hidden layer, $h_{k}={\left[\begin{array}{cccc} h_{k,1} & h_{k,2} & \cdots & h_{k,m} \end{array}\right]}^{T}$ and $h_{k,j}=\varphi \left({∥x_{k}-c_{j}∥}^{2}\right)$.

A Gaussian function is selected as the neuron function of the hidden layer, expressed as:

(23) $$\varphi \left(x\right)=e^{-\frac{x}{\sigma^{2}}}$$

The gradient descent method is adopted to solve the network parameters. Firstly, an energy consumption function is established as:

(24) $$E=\frac{1}{2}\sum_{t=1}^{s}\sum_{i=1}^{n}{\left(y_{i,t}-{\hat{y}}_{i,t}\right)}^{2}$$

where $y_{i,t}$ is the true value, ${\hat{y}}_{i,t}$ is the estimated value, *s* is the number of samples, and *n* is the number of results. According to the extremum principle, the gradient of the energy consumption function is found, on the negative side of which the network parameters are traversed until the energy consumption function meets the error precision, and the optimal estimated network parameters are obtained.

Firstly, the increment of the weight factor in the direction of its negative gradient is obtained as:

(25) $$\Delta \omega_{p}=-\alpha \nabla_{\omega_{p}}E=\alpha \sum_{t=1}^{s}\sum_{i=1}^{n}\left(y_{i,t}-{\hat{y}}_{i,t}\right)\nabla_{\omega_{p}}{\hat{y}}_{i,t}$$

where α is the learning speed of the weight factor. The gradient of the estimated value is expressed as:

(26) $$\nabla_{\omega_{p}}{\hat{y}}_{i,t}=\nabla_{\omega_{p}}\left[\omega_{i}^{T}h_{t}\right]=\delta_{ip}h_{t}$$

(27) $$\delta_{ip}=\left\{\begin{array}{c} 1\;\;\;\;\;\;i=p\\ 0\;\;\;\;\;i\neq p \end{array}\right.$$

Similarly, the learning speed of the center points is set as λ, and the increment on negative gradient is expressed as:

(28) $$\Delta c_{q}=-\lambda \nabla_{c_{q}}E=\lambda \sum_{t=1}^{s}\sum_{i=1}^{n}\left(y_{i,t}-{\hat{y}}_{i,t}\right)\nabla_{c_{q}}{\hat{y}}_{i,t}$$

(29) $$\nabla_{c_{q}}{\hat{y}}_{i,t}=\nabla_{c_{q}}\left(\sum_{j=1}^{m}\omega_{i,j}h_{t,j}\right)=\omega_{i,q}\nabla_{c_{q}}h_{t,q}$$

(30) $$\nabla_{c_{q}}h_{t,q}=\nabla_{c_{q}}\left(e^{-\frac{{∥x_{t}-c_{q}∥}^{2}}{\sigma^{2}}}\right)=\frac{2}{\sigma^{2}}e^{-\frac{{∥x_{t}-c_{q}∥}^{2}}{\sigma^{2}}}∥x_{t}-c_{q}∥$$

The extended constant σ of the radial basis function is chosen as follows:

(31) $$\sigma =\frac{d_{\max}}{\sqrt{2m}}$$

where $d_{\max}$ is the maximum distance of the center points.

In summary, the training steps of RBFNN parameters by gradient descent method are as follows:

(1) Set the learning speed parameters α and λ, initialize the weight coefficient $\omega_{i}=0$, and select the central points $c_{j}$ randomly, where i=1,2,⋯n, j=1,2,⋯m

(2) Calculate the initial response:

• $h_{t,j}=\varphi \left({∥x_{t}-c_{j}∥}^{2}\right)\;\forall t,j$;
• $h_{t}={\left[\begin{array}{cccc} h_{t,1} & h_{t,2} & \cdots & h_{t,m} \end{array}\right]}^{T}\;\forall t$;
• ${\hat{y}}_{i,t}=\omega_{i}^{T}h_{t}\;\forall i,t$.

(3) Calculate the energy consumption function:

$E=\frac{1}{2}\sum_{t=1}^{s}\sum_{i=1}^{n}{\left(y_{i,t}-{\hat{y}}_{i,t}\right)}^{2}$

Furthermore, let $E_{old}=E$

(4) Update the parameters:

• $\Delta \omega_{i}=\alpha \sum_{t=1}^{s}\left(y_{i,t}-{\hat{y}}_{i,t}\right)h_{t}\;\forall i,t$;
• $\omega_{i}\;\leftarrow \;\omega_{i}+\Delta \omega_{i}\;\;\forall i$;
• $\Delta c_{j}=-\frac{2\lambda }{\sigma^{2}}\sum_{t=1}^{s}\sum_{i=1}^{n}\left(y_{i,t}-{\hat{y}}_{i,t}\right)\omega_{i,j}e^{-\frac{{∥x_{t}-c_{j}∥}^{2}}{\sigma^{2}}}∥x_{t}-c_{j}∥\;,\;\forall j$;
• $c_{j}\leftarrow c_{j}+\Delta c_{j}$.

(5) Calculate the current response:

• $h_{t,j}=e^{-\frac{{∥x_{t}-c_{j}∥}^{2}}{\sigma^{2}}}\;\forall t,j$;
• $h_{t}={\left[\begin{array}{cccc} h_{t,1} & h_{t,2} & \cdots & h_{t,m} \end{array}\right]}^{T}\;\forall t$;
• ${\hat{y}}_{i,t}=\omega_{i}^{T}h_{t}\;\forall i,t$.

(6) Calculate the current residual and compare:

$E_{now}=\frac{1}{2}\sum_{t=1}^{s}\sum_{i=1}^{n}{\left(y_{i,t}-{\hat{y}}_{i,t}\right)}^{2}$;

If $\frac{E_{old}-E_{now}}{E_{old}}>\varepsilon$, return to step (3) and repeat;

If $\frac{E_{old}-E_{now}}{E_{old}}<\varepsilon$, finish training.

Here, ε is the selected network training accuracy.

Based on a measurement result, the trained RBFNN is used to fit the measurement error, which is subtracted from the measurement result, and a more accuracy measurement result ${\left[\begin{array}{cc} \alpha_{RBFNN} & \beta_{RBFNN} \end{array}\right]}^{T}$ can be obtained.

It is crucial to emphasize that the dataset used for training should cover the working range of the equipment; otherwise, the trained RBFNN will only exhibit high accuracy within a local range and its precision will decrease or even result in errors beyond that scope.

## 4. Simulations and Experiments

### 4.1. Simulations

The LS-based error compensation method need a certain number of error samples to construct overdetermined equation to solve the error parameters, the RBFNN-based error compensation method also need a certain number of error samples to train the approximation network. In order to verify the superiority of the different compensation methods, the same sample space is selected for the two methods. The azimuth and pitch rotation ranges of the equipment are $\left[-90^{\circ },90^{\circ }\right]$ and $\left[-20^{\circ },80^{\circ }\right]$, respectively. Starting from $-90^{\circ }$ and $-20^{\circ }$, an azimuth value and a pitch value are selected at every $5^{\circ }$ interval, resulting in a total of 37×21 values being chosen, which serve as measurement or input values. The error values obtained from the corresponding 37×21 values of simulation results in Figure 12 and Figure 13 are utilized as measurement errors or target values, so a common sample is established. It can be observed that the selected sample covers the operational range of the equipment and the corresponding errors are also randomly distributed, so the selected sample space is universal.

When training the RBFNN with the above sample, the initial weight coefficient vector $\omega_{i}=0$, and the initial center points are taken as azimuth and pitch values of 37×21 of the sample. The extended constant is set to the maximum distance between center points, σ=5.22. The LS-based error compensation method does not need to set any parameters when calculating the optimal error parameters with the above sample.

**Figure 15:**
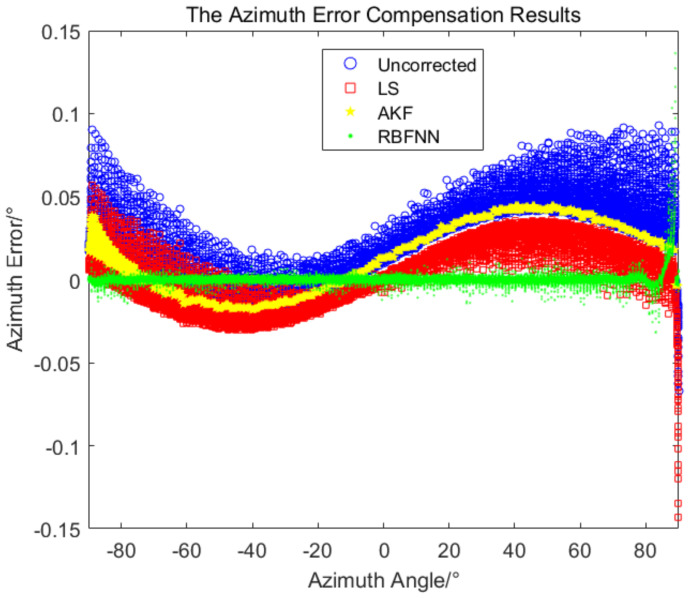
The comparison of the azimuth measurement accuracy for the three methods.

**Figure 16:**
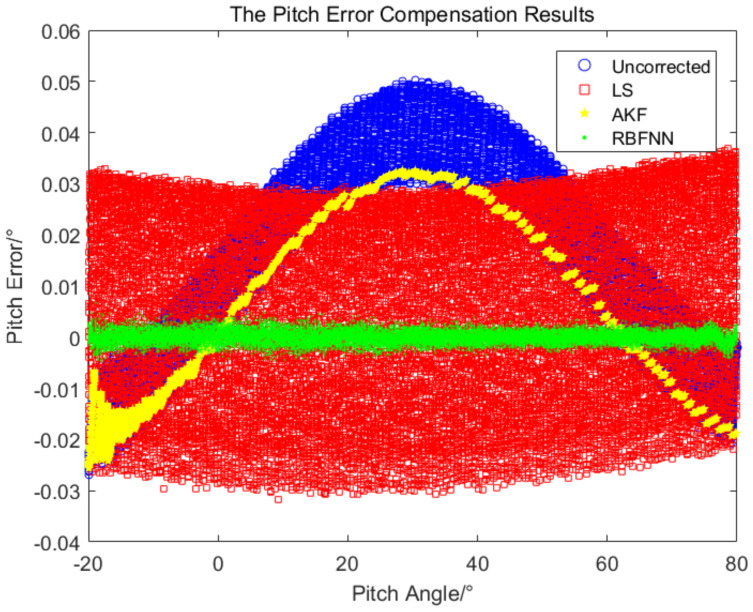
The comparison of the pitch measurement accuracy for the three methods.

The AKF-based method to correct the measurement error does not require sample space, the initial process noise and measurement noise are set as 0.1 and 0.012, respectively, and the window smoothing parameter is set as m=3. The three error compensation methods above are used for simulation comparison, and the results are shown in Figure 15 and Figure 16.

As can be seen from the simulation results in Figure 15 and Figure 16, the AKF filtering algorithm filtered the burr part of the measured data and did not filter the absolute error of the measurement data, the measured data become relatively smooth, and the variation trend of the modified error is consistent with the original error. The LS-based error compensation method is a linear regression of the nonlinear error. As can be seen from the simulation results, the error after regression is distributed symmetrically on both sides of the coordinate axis *x*, but the fluctuation of the errors is still relatively large. The RBFNN-based error compensation method mapped the linear input into the nonlinear space, the error variation was better fitted, and the error is a narrow band after compensation.

Table 2 shows the statistical results of the three methods. The standard deviations of the results of the LS-based method and the AKF-based method are basically the same, and the standard deviations of the azimuth and pitch errors are about $0.02^{\circ }$. The resulting mean of the LS method is better than that of the AKF method, and for the azimuth error mean, the result of the LS method is better than that of the AKF method by one order of magnitude. In the error compensation results based on RBFNN, the mean and standard deviation are more than one order of magnitude better than the results of other two methods, in which the azimuth error standard deviation is $0.005^{\circ }$ and the pitch error standard deviation is $0.001^{\circ }$.

The accuracy of the error compensation method based on RBFNN depends on the number of neurons. Two additional samples were selected for comparison, one with a sample size of 66 ($18^{\circ }$ equal spacing for azimuth and $20^{\circ }$ equal spacing for pitch, and the corresponding error values) and the other with a sample size of 209 ($10^{\circ }$ equal spacing for azimuth and pitch, and the corresponding error values). This two new samples were used to train a new RBFNN, respectively, and the error compensation were carried out with the corresponding new RBFNN. The results are compared as follows.

**Figure 17:**
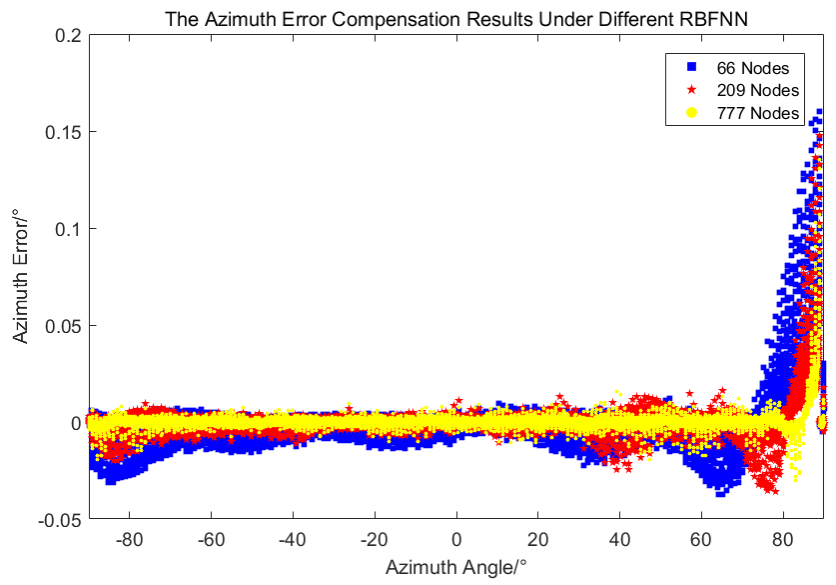
The azimuth error after compensation under RBFNN with different nodes.

**Figure 18:**
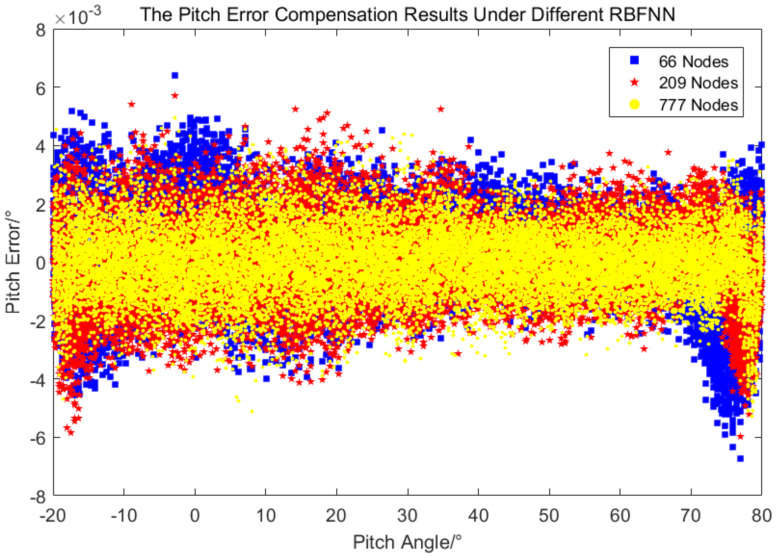
The pitch error after compensation under RBFNN with different nodes.

It can be seen from Figure 17 and Figure 18 that the errors after compensation by three new RBFNN are distributed symmetrically on both sides of the coordinate axis *x*, and the error fluctuation is larger as the number of neurons decreases. Table 3 shows the statistical results of three new RBFNN, the results of the 777-node RBFNN are better than those of the 209-node RBFNN, but both the mean and standard deviation are in the same order of magnitude and the difference is not large. The pitch error standard deviation of the 66-node RBFNN is the same order of magnitude as the other two, but the azimuth error standard deviation is one order of magnitude larger than the two other. Considering the convenience and economy of engineering application, the 209-node RBFNN is recommended for measuring error compensation.

On the other hand, the three methods of error compensation or correction have different requirements for computational power. Clearly, the AKF-based method requires the minimum computational power, followed by LS-based method, while the RBFNN-based method has the highest demand for computational power and it increases significantly with an increase in the number of neurons. Fortunately, RBFNN training can be conducted on a host computer and then transfered to an embedded system to achieve real-time error compensation.

### 4.2. Experiments

A two-axis electro-optical measurement equipment and a helicopter with GPS were used to build a test environment. The equipment consists of a spherical pitch package and a cylindrical azimuth mechanism, with dimensions of 1.2 m in height, 0.4 m in diameter, and the total weight is 125 kg. Moreover, the visible light camera, infrared camera, laser range finder, laser sensor, and fiber optic gyroscopes are integrated in the pitch package using inertial stabilization principle for LOS stability. The visible and infrared sensors enable imaging, tracking, and angle measurement of the target during both day and night conditions, while the laser range finder allows for radial distance measurement of the target. Under favorable weather conditions, the equipment has an integrated range exceeding 12 km. The helicopter, acting as the target, is capable of hovering or flying along a prescribed path. A GPS positioning device was installed on the helicopter, and the positioning accuracy of the GPS positioning device is millimeter accuracy level. During the test, the designed radial distance between the helicopter and the equipment is more than 3 km, so the angle value obtained by GPS conversion is of the order of $10^{-5}$, which can be used as the true value of the test. In the experiments, the spatial azimuth and pitch of the helicopter were measured by the two-axis electro-optical measurement equipment, and they are taken as measured value; the azimuth and pitch angles relative to the two-axis electro-optical measurement equipment were calculated by using the GPS of the helicopter, and they are taken as the true value.

**Figure 19:**
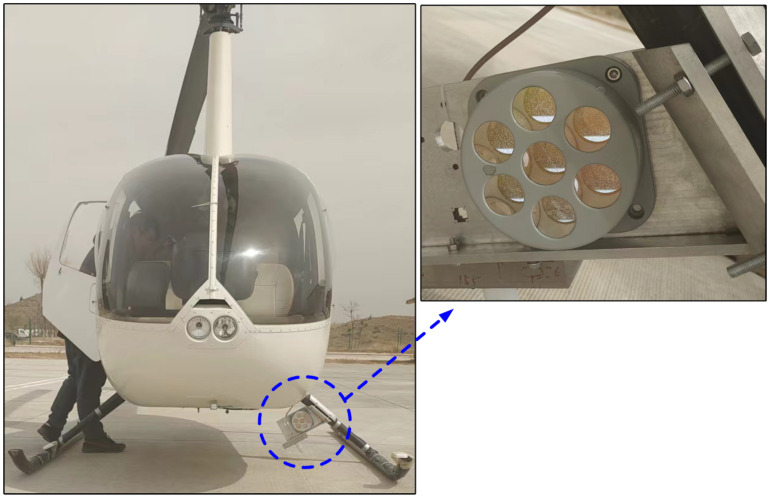
The helicopter fitted with the cooperative target.

The target is tracked and measured by the method of target image extraction, but the movement of the target makes its image characteristics change, which leads to the instability of the tracking point and brings unnecessary errors to the measurement results. In order to keep the tracking point fixed, an optical cooperative target was installed on the helicopter landing gear and was taken as a fixed tracking measurement point, as shown in Figure 19.

**Figure 20:**
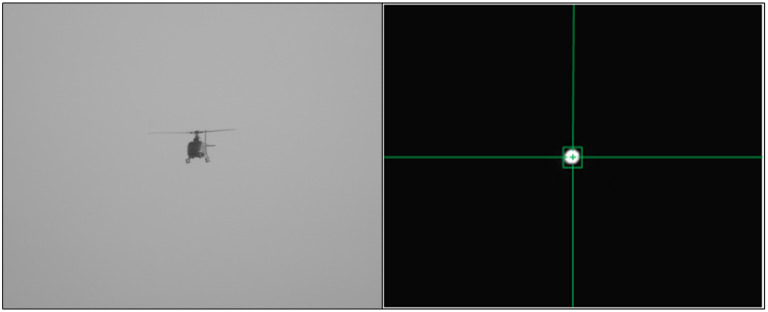
Flight test and target image point.

The visible camera of the equipment was used to capture the helicopter, when the helicopter was tracked steadily by the visible light image, the laser sensor emitted the laser pulses to the helicopter, and the laser echo was imaged as a bright spot by the short-wave infrared sensor, and then it was converted into tracking and measurement of the bright spot, as shown in Figure 20. It can be seen that the short-wave infrared sensor only images the laser reflected back by the optical cooperative target, and the image was a bright spot; thus, the problem of unstable tracking points was solved.

For the equipment, the azimuth rotation range is $\left[-90^{\circ },90^{\circ }\right]$, the pitch rotation range is $\left[-20^{\circ },80^{\circ }\right]$, and the output frequency of the measured data is 20 Hz. Within the rotation range of azimuth and pitch, the flight path of the helicopter is planned to cover the measuring range of the equipment as much as possible. At the beginning of the experiment, the equipment and the helicopter were time-matched, and the true value was calculated at a rate of 20 Hz by using the GPS, so the measured value and the true value are obtained at the same time. It should be noted that there was a fixed installation distance between the optical cooperative target and the helicopter GPS antenna, considering this installation distance, the GPS measurement value was converted to the installation position of the optical cooperative target.

According to the above experiment method, a large number of measured values and true values are obtained. If it was used to train the RBFNN directly, it will not only require a large amount of computation, but also may cause the calculation not converge. Thus, the values were obtained at an equal interval of $8^{\circ }$ in the range of azimuth and pitch, respectively, and a total of 21 × 11 azimuth and pitch values are obtained. In the vicinity of each azimuth and pitch value obtained, the nearest measurement values were found, and the true values were selected at the corresponding time, a total of 231 samples are obtained. Based on the selected samples, a new RBFNN was trained by the above training method.

**Figure 21:**
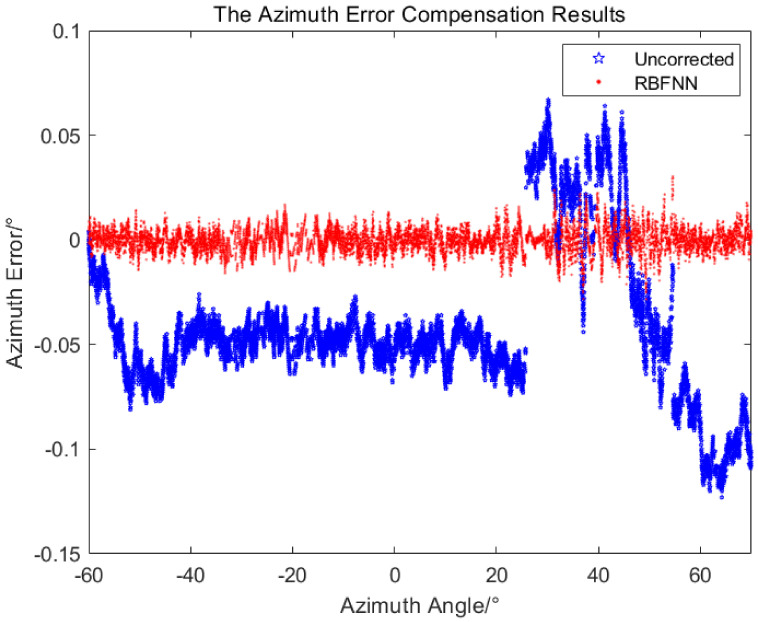
The azimuth errors in the experiment.

**Figure 22:**
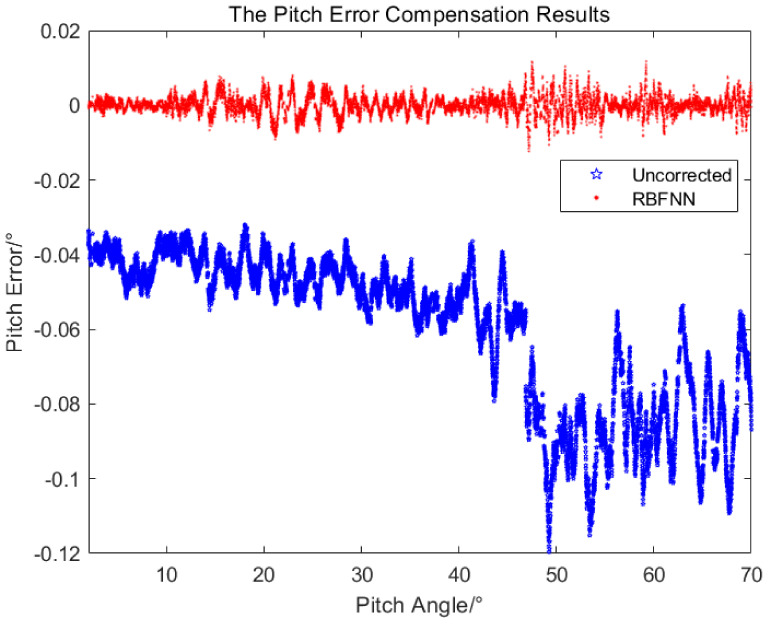
The pitch errors in experiment.

Using the obtained RBFNN, the measured value of the equipment was output after error compensation in real time. For the flight experiment of the helicopter, the measured value after error compensation by the RBFNN and the measured value without error compensation were compared with the true value, respectively, and the measured errors of the azimuth and pitch are shown in Figure 21 and Figure 22.

It can be seen from Figure 21 and Figure 22, the measurement errors without error compensation deviate greatly from $0^{\circ }$, while the measurement errors after error compensation by RBFNN oscillate around $0^{\circ }$ with relative small error band, the measurement accuracy is significantly improved. As shown in Table 4, after adopting the RBFNN-based error compensation method, the average values of the azimuth measurement errors and the pitch measurement errors are $-0.0003^{\circ }$ and $0.00003^{\circ }$, and the standard deviations are $0.006^{\circ }$ and $0.003^{\circ }$, respectively. With 95% confidence, the corresponding error confidence intervals are $\left[-0.0004^{\circ },-0.0002^{\circ }\right]$ and $\left[-0.00003^{\circ },0.00009^{\circ }\right]$, respectively. However, without the RBFNN-based error compensation method, the average values of the azimuth and pitch errors measured are $-0.04^{\circ }$ and $-0.06^{\circ }$, the standard deviations are $0.04^{\circ }$ and $0.02^{\circ }$. With 95% confidence, the corresponding error confidence intervals are $\left[-0.043^{\circ },-0.041^{\circ }\right]$ and $\left[-0.0604^{\circ },-0.0596^{\circ }\right]$, respectively. The application of the RBFNN-based error compensation method significantly enhanced the accuracy of the target measurement.

## 5. Conclusions

In order to enhance the target measurement accuracy of two-axis electro-optical measurement equipment, an active compensation technology for target measurement error was proposed. Firstly, various error sources that affect the target measurement accuracy were analyzed, and an error transfer model was established. Through simulation analysis, it was found that the target measurement errors exhibit a spatial free-form surface with random irregularities. Subsequently, three methods for error compensation or correction were proposed: the LS-based error compensation method, AKF-based error compensation method, and RBFNN-based error compensation method. Simulation analysis revealed that the RBFNN-based error compensation method can accurately approximate the error distribution and achieve more precise error correction, thereby significantly improving the target measurement accuracy. In addition, the influence of the number of neurons on the accuracy of the RBFNN-based error compensation method was analyzed and discussed. Finally, the experimental results demonstrated the engineering usability of the proposed method, and it greatly improved the target measurement accuracy of the two-axis electro-optical measurement equipment.

Although the proposed active error compensation technology improved the target measurement accuracy of a specific electro-optical measurement equipment, it did not consider the influence on the error sources in temperature changes, usage scenario changes, and so on, so there may be some limitations in the application of this method. Based on this, it will be a future research direction to propose an error compensation technique that can adapt to temperature changes, usage scenario changes, and so on. 

## Figures and Tables

**Table 1:** The statistical errors of the two-axis electro-optical measurement equipment.

Number	Items		Value${/}^{\circ }$	Comment
1	Target image extraction error	$\sqrt{\Delta m{d_{y}}^{2}+\Delta m{d_{z}}^{2}}$	0.006	Random distribution
2	Perpendicular error of the sensor installation	$\Delta p_{p}$	0.005	Uniform distribution
3	Pitch angle measurement error	Δpa	0.002	Random distribution
4	Pitch axis wobble	$\sqrt{\Delta {p_{x}}^{2}+\Delta {p_{z}}^{2}}$	0.004	Random distribution
5	Pitch horizontal zero error	$\Delta p_{h}$	0.009	Uniform distribution
6	Perpendicular error between pitch and azimuth axes	Δap	0.0075	Uniform distribution
7	Azimuth angle measurement error	Δaa	0.002	Random distribution
8	Azimuth axis wobble	$\sqrt{\Delta {a_{x}}^{2}+\Delta {a_{y}}^{2}}$	0.002	Random distribution
9	Azimuth aero error	Δae	0.0063	Uniform distribution
10	Perpendicular error of equipment installation	Δape	0.0085	Uniform distribution

**Table 2:** The error compensation results.

Items	Azimuth		Pitch	
	**Average${/}^{\circ }$**	**Standard Deviation${/}^{\circ }$**	**Average${/}^{\circ }$**	**Standard Deviation${/}^{\circ }$**
Uncorrected	0.018983	0.023454	0.016161	0.019025
LS	0.003527	0.019322	0.005762	0.018193
AKF	0.013641	0.021185	0.008187	0.017594
RBFNN	0.000262	0.004924	0.000028	0.001173

**Table 3:** The measurement error compensation results under RBFNN with different nodes.

Items	Azimuth		Pitch	
	Average${/}^{\circ }$	Standard Deviation${/}^{\circ }$	Average${/}^{\circ }$	Standard Deviation${/}^{\circ }$
66 Nodes	0.000387	0.011074	0.000069	0.001376
209 Nodes	0.000553	0.007510	0.000125	0.001218
777 Nodes	0.000262	0.004924	0.000028	0.001173

**Table 4:** The experiment results.

Items	Azimuth		Pitch	
	Average${/}^{\circ }$	Standard Deviation${/}^{\circ }$	Average${/}^{\circ }$	Standard Deviation${/}^{\circ }$
Uncorrected	−0.042048	0.035604	−0.060048	0.020439
RBFNN	−0.000266	0.005731	0.000034	0.002516

## Data Availability

Data are contained within the article.

## Abbreviations

The following abbreviations are used in this manuscript:

LOS	Line of sight
RBFNN	Radial basis function neural network
LS	Least square
AKF	Adpative Kalman filter

## References

[1] Liu Y., Peng Y., Yan J. (2022). Effect of the Azimuth Axis Tilt Error on the Tracking Performance of a Solar Dish Concentrator System. Energies.

[2] Li P., Zhao R., Luo L. (2020). A Geometric Accuracy Error Analysis Method for Turn-Milling Combined NC Machine Tool. Symmetry.

[3] Wang L., Li M., Yu G. (2023). A Novel Error Sensitivity Analysis Method for a Parallel Spindle Head. Robotics.

[4] Fang W., Tian X. (2021). Geometric error sensitivity analysis for a 6-axis welding equipment based on Lie theory. Int. J. Adv. Manuf. Technol..

[5] Li Q., Wang W., Jiang Y., Li H., Zhang J., Jiang Z. (2018). A sensitivity method to analyze the volumetric error of five-axis machine tool. Int. J. Adv. Manuf. Technol..

[6] Zhou B., Wang S., Fang C., Sun S., Dai H. (2017). Geometric error modeling and compensation for five-axis CNC gear profile grinding machine tools. Int. J. Adv. Manuf. Technol..

[7] Yang J., Ding H. (2016). A new position independent geometric errors identification model of five-axis serial machine tools based on differential motion matrices. Int. J. Mach. Tools Manuf..

[8] Yin S., Zhou H., Peng Y., Ju X. (2023). Dual quaternion-based kinematic modeling for decoupling identification of geometric errors of rotary axes in five-axis platforms. Precis. Eng..

[9] Fan J., Tao H., Pan R., Chen D. (2020). An approach for accuracy enhancement of five-axis machine tools based on quantitative interval sensitivity analysis. Mech. Mach. Theory.

[10] Feng X., Sun H., Lv T., Zhang Y. (2018). Kinematic analysis of a PPPR spatial serial mechanism with geometric errors. Proc. Inst. Mech. Eng. Part C J. Mech. Eng. Sci..

[11] Li J., Zhao Y., Tang Q., Sun W., Yuan F., Lu X. (2021). Conceptual design and error analysis of a cable-driven parallel robot. Robotica.

[12] San H., Ding L., Zhang H., Wu X. (2023). Error Analysis of a New Five-Degree-of-Freedom Hybrid Robot. Actuators.

[13] Guo S., Mei X., Jiang G. (2019). Geometric accuracy enhancement of five-axis machine tool based on error analysis. Int. J. Adv. Manuf. Technol..

[14] Guo S., Jiang G., Mei X. (2017). Investigation of sensitivity analysis and compensation parameter optimization of geometric error for five-axis machine tool. Int. J. Adv. Manuf. Technol..

[15] Ding S., Huang X., Yu C., Wang W. (2016). Actual inverse kinematics for position-independent and position-dependent geometric error compensation of five-axis machine tools. Int. J. Mach. Tools Manuf..

[16] Wu C., Fan J., Wang Q., Chen D. (2018). Machining accuracy improvement of non-orthogonal five-axis machine tools by a new iterative compensation methodology based on the relative motion constraint equation. Int. J. Mach. Tools Manuf..

[17] Zhou D., Gong S., Wang Z., Li D., Lu H. (2020). Error analysis based on error transfer theory and compensation strategy for LED chip visual localization systems. J. Intell. Manuf..

[18] He Y., Zhang Y., Feng X., Deng S., Wang Z. (2023). Pointing Error Correction for a Moving-Platform Electro-Optical Telescope Using an Optimized Parameter Model. Sensors.

[19] Peng C., He D., Wang Y., Wang Z., Huang Y., Zhang T., Liu X., Wang Q., Ma H. (2023). Pointing-error correction of optical communication terminals on motion platforms using a parameter model and kernel weight function estimation. Appl. Opt..

[20] Liang X., Zhou J., Ma W. (2019). Method of distortion and pointing correction of a ground-based telescope. Appl. Opt..

[21] Zhao H., Li S., Jiang T., Hong Y., Ma Z. (2023). Modeling and digital calibration for the mirror normal pointing error of the 2D scanning reflector. Appl. Opt..

[22] Wan M., Zheng R., Zhao H., Yu L. (2023). Accuracy improvement of multi-view 3D laser scanning measurements based on point cloud error correction and global calibration optimization. Opt. Express.

